# Circulating microRNAs Signature for Predicting Response to GLP1-RA Therapy in Type 2 Diabetic Patients: A Pilot Study

**DOI:** 10.3390/ijms22179454

**Published:** 2021-08-31

**Authors:** Caterina Formichi, Daniela Fignani, Laura Nigi, Giuseppina Emanuela Grieco, Noemi Brusco, Giada Licata, Claudia Sabato, Elisabetta Ferretti, Guido Sebastiani, Francesco Dotta

**Affiliations:** 1 Diabetes Unit, Department of Medicine, Surgery and Neurosciences, University of Siena, 53100 Siena, Italy; catefo@libero.it (C.F.); dfignani@gmail.com (D.F.); launigi@gmail.com (L.N.); giusy.grieco.90@gmail.com (G.E.G.); noemibrusco91@gmail.com (N.B.); giadalicata.92@gmail.com (G.L.); sebastianiguido@gmail.com (G.S.); 2 Fondazione Umberto Di Mario, c/o Toscana Life Sciences, 53100 Siena, Italy; 3 Department of Experimental Medicine, Sapienza University of Rome, 00161 Rome, Italy; claudia.sabato@uniroma1.it (C.S.); elisabetta.ferretti@uniroma1.it (E.F.); 4 Tuscany Centre for Precision Medicine (CReMeP), 53100 Siena, Italy

**Keywords:** microRNAs, GLP1-RA, type 2 diabetes, obesity, personalized medicine

## Abstract

Type 2 diabetes (T2D) represents one of the major health issues of this century. Despite the availability of an increasing number of anti-hyperglycemic drugs, a significant proportion of patients are inadequately controlled, thus highlighting the need for novel biomarkers to guide treatment selection. MicroRNAs (miRNAs) are small non-coding RNAs, proposed as useful diagnostic/prognostic markers. The aim of our study was to identify a miRNA signature occurring in responders to glucagon-like peptide 1 receptor agonists (GLP1-RA) therapy. We investigated the expression profile of eight T2D-associated circulating miRNAs in 26 prospectively evaluated diabetic patients in whom GLP1-RA was added to metformin. As expected, GLP1-RA treatment induced significant reductions of HbA1c and body weight, both after 6 and 12 months of therapy. Of note, baseline expression levels of the selected miRNAs revealed two distinct patient clusters: “high expressing” and “low expressing”. Interestingly, a significantly higher percentage of patients in the high expression group reached the glycemic target after 12 months of treatment. Our findings suggest that the evaluation of miRNA expression could be used to predict the likelihood of an early treatment response to GLP1-RA and to select patients in whom to start such treatment, paving the way to a personalized medicine approach.

## 1. Introduction

Type 2 diabetes (T2D) is a heterogeneous disease with varying clinical presentation and disease progression, in which genetic and environmental factors contribute to progressive loss of adequate insulin secretion, frequently on the background of insulin resistance [1]. Diabetes prevalence is rapidly increasing, becoming a global epidemic and representing a major health and socioeconomic issue worldwide [2,3]. According to the International Diabetes Federation (IDF), in 2019 approximately 9.3% of adults aged 20–79 years suffered from diabetes worldwide, and the number of diabetic patients is predicted to reach 700 million by 2045 [4]. Diabetic patients are at risk for developing micro and macrovascular complications and, indeed, T2D is associated with high morbidity and mortality [1], being one of the leading causes of cardiovascular disease, end stage renal disease and blindness.

One of the main challenges faced by clinicians in the treatment of T2D is to select the most appropriate therapy for each patient, one that provides the best efficacy among the many options available. Unfortunately, a significant proportion of diabetic patients fail to achieve optimal glycemic control and are therefore at higher risk of developing chronic disease complications. It is well demonstrated that glycemic control is fundamental to diabetes management, and achieving HbA1c targets <7% (53 mmol/mol) has been shown to reduce long-term complications in both type 1 and type 2 diabetes [5,6]. The benefits of an adequate glycemic control are more pronounced when the glycemic target is reached in the initial phase of the disease, with enduring effects in long term follow-up (legacy effect) [5].

In recent years, the availability of new classes of drugs has radically changed the landscape of T2D treatment, expanding therapeutic options. However, it is not always easy for clinicians to choose from such a wide variety of options. The growing uncertainty regarding the proper selection of antidiabetic agents faced by clinicians prompted the American Diabetes Association (ADA) and the European Association for the Study of Diabetes (EASD) to recommend application of individualized treatment and precision medicine for diabetes [1,7,8,9]. Indeed, the shift from a ‘one-size fits all’ approach to personalized medicine, will allow adequate glycemic control to be achieved in a short time, with significant benefits on long-term complications and less discomfort for the patient. The individualized approach in drug selection might be facilitated by pharmacogenetics [3]. In recent years, significant progress was made in this field, prompting researchers to suggest the use of pharmacogenetics to guide drug selection. For example, single nucleotide polymorphisms (SNPs) in SLC22A1 and SLC2A2 genes have been related to better efficacy of metformin treatment [9,10,11], whereas other genetic variants, such as a reduced-function alleles of the OCT1 gene, may be associated to gastrointestinal side effects of metformin [9,12,13]. Similar data also highlighted the influence of genetic variants on treatment response to thiazolidinediones (TZDs) [14], sulphonylureas and metaglinides [10]. Available evidence also suggests that gene polymorphisms may mediate the response to novel glucose-lowering drugs. In a recent review, Rathmann and colleagues summarized available findings regarding the role of pharmacogenetics in the response to dipeptidyl peptidase 4-inhibitors (DPP4i), glucagon-like peptide-1 receptor agonists (GLP1-RA) and sodium-glucose cotransporter 2 inhibitors (SGLT2i) treatments [10]. As an example, genetic polymorphisms in GLP-1 receptor gene seem to influence the interindividual differences in response to both DPP4i and GLP1-RA treatment [10,15,16], with some variants associated with poorer glycemic response and others associated with higher HbA1c reduction. In particular, the GLP1R rs6923761 A allele has been related to better HbA1c reduction and weight loss after liraglutide therapy, while the T allele of GLP1R rs10305420 was associated with lower HbA1c and weight reduction in exenatide-treated patients [10]. CNR1, TCF7L2 and SORCS1 variants are also reported to influence GLP1-R response [10,16]. However, despite advances in pharmacogenetic approach, clinical application is hindered by heterogeneity of T2D, with many interacting genetic and environmental factors determining disease progression and treatment success. Thus, genetic information should be combined with clinical and molecular markers to better stratify T2D patients and to guide clinical management [10].

The main obstacle to personalized medicine is the lack of reliable and accurate biomarkers to identify the best treatment option for each patient at an early stage. With this in mind, microRNAs (miRNAs) have been suggested as biomarkers to predict the therapeutic efficacy of diabetes treatments. MiRNAs are endogenous small non-coding RNAs (19–24 nucleotides) that negatively modulate gene expression at post transcriptional level, involved in many cellular processes such as development, cell proliferation and survival, differentiation and apoptosis [17,18]. MiRNAs are valuable candidates as biomarkers given their high stability and easy detection in biological fluids [18]. The potential use of miRNAs as biomarkers of therapeutic efficacy is currently under investigation in T2D patients treated with different therapeutic options. For instance, Demirsoy and colleagues demonstrated an altered expression profiles of 13 microRNAs, which were found significantly downregulated following three months of metformin treatment in T2D patients [19]. In a recent study on prospectively evaluated T2D patients, in whom sitagliptin was added to background metformin therapy, it was shown that high circulating levels of miR-378 appear to be a negative predictor of response to sitagliptin, while miR-126–3p and miR-223 seem to be markers of response to the drug [20]. In another study, circulating miRNAs were evaluated to predict individual response to TZDs. Authors identified four miRNAs positively associated with drug response (miR-20b-5p, miR-214-3p, miR-22-3p and miR-486-5p) and two miRNAs negatively related to TZD treatment outcome (miR-21-5p, miR-320a). Moreover, miR-320a and miR-486-5p identified TZD responders among the insulin resistant subjects [21]. Nunez Lopez and colleagues demonstrated that baseline levels of miR-145-5p and miR-29c-3p, together with HbA1c levels, could predict the response to short-term intensive insulin therapy [22].

No miRNA has yet been described as a biomarker for predicting response to GLP1-RA treatment in diabetes mellitus. Given the role of miRNAs as promising biomarkers of drug response in T2D, the aim of our study was to evaluate the expression of a set of circulating microRNAs in plasma of prospectively evaluated T2D patients before and after 6 and 12 months of therapy with GLP1-RA (liraglutide and dulaglutide), in combination with metformin, to assess whether the baseline levels of these circulating miRNAs could predict the therapeutic outcome in terms of glycemic control and weight loss.

Based on a literature search, we selected the following eight microRNAs on the basis of the previously demonstrated association between their expression levels and beta-cell function and/or glucose metabolism in T2D: miR-24-3p [23,24], miR-126-3p [20,23,25,26,27,28,29,30,31], miR-21-5p [23,29,32,33], miR-15a-5p [23,27,34], miR-223-3p [20,23,27], miR-378-3p [20], miR-375-3p [19,33] and miR-146a-5p [33,35]. As an example, miR-126-3p has been shown to be reduced in T2D [23] and pre-diabetes [23,25,26,28], while miR-146a-5p expression increased in T2D and pre-diabetic patients compared with normoglycemic subjects [36,37], and was suggested to play a pathogenic role in diabetes [37]. MiR-126-3p, miR-146a-5p and miR-21-5p have been involved in the insulin signaling pathway by targeting different molecules of the insulin signaling cascade (respectively IRS1 and 2, PTPN1, PTEN) in several cellular types, such as beta-cells to hepatocytes, adipocytes and myotubes [17]. Additional miRNAs were reported to control metabolic pathways; indeed, high expression of miR-375-3p has been associated with β cell dysfunction and insulin secretion [38].

Among the eight miRNAs selected in the present work, some have been described as differentially expressed in insulin-sensitive tissues involved in the pathogenesis of the metabolic syndrome and insulin resistance (i.e., pancreatic islets, liver, adipose tissue, skeletal muscle and endothelium). For example, miR-375-3p is enriched in the endocrine pancreas and is fundamental to β cell survival and function, being involved in glucose-induced insulin secretion [17]. In liver tissue from diet-induced insulin resistant mice, the downregulation of several miRNAs, among which miR-378a-3p and miR-223-3p have been described, with potential involvement in the insulin signaling pathway and hepatic lipids synthesis. In particular, hepatic miR-223 is involved in cholesterol synthesis and efflux [39]. As a matter of fact, increased expression of miR-223-3p, alongside with miR-146a-5p and miR-21-5p, has been shown in hepatic and cardiac tissues in animal models of hyperlipidemia. miR-146a-5p has also been found increased in human atherosclerotic plaques and increased circulating levels of miR-146a-5p characterize patients with acute coronary syndrome [40]. miR-223 has also been involved in adipose tissue dysfunction, preceding T2D onset. Indeed, under systemic inflammatory conditions, pro-inflammatory cytokine TNFα promotes intracellular accumulation of miR-223 in preadipocytes, which in turn impairs their differentiation [41]. Upregulation of miR-146a-5p and miR-21a-5p has been demonstrated in pancreatic islets under inflammatory conditions, and may affect the expression of islet-specific transcription factors, contributing to islet dysfunction [42]. 

Against this background, highlighting the involvement of this set of miRNAs in central and peripheral regulation of metabolic pathways, we presently analyzed them at baseline in plasma of T2D patients being subjected to GLP1-RA treatment and followed-up for a period of up to 12 months. We showed that a subset of these selected circulating miRNAs could be used to predict treatment outcome, thus guiding T2D patients towards a personalized medicine approach.

## 2. Results

### 2.1. GLP1-RA Treatment Outcomes in T2D Patients

**Figure 1:**
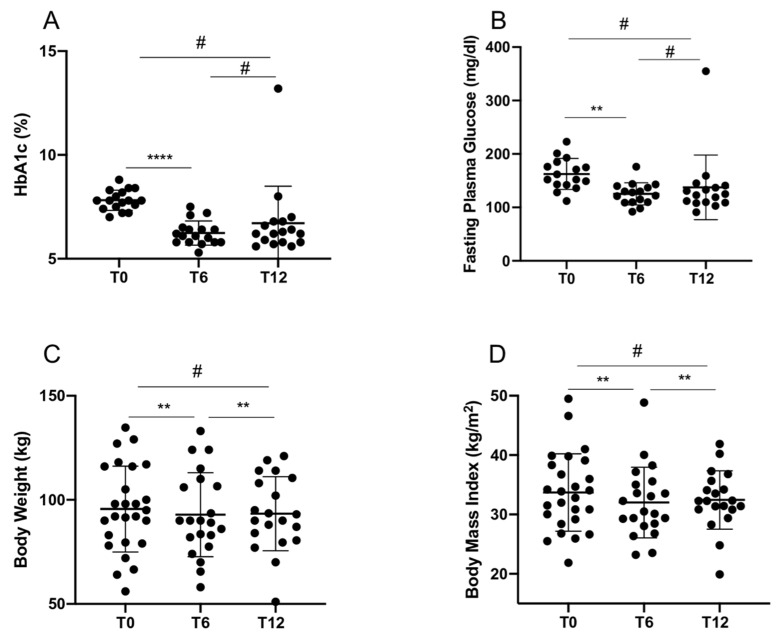
Comparison of HbA1c values (**A**), fasting plasma glucose (**B**), body weight (**C**) and body mass index (**D**) between baseline (T0) and after 6 (T6) and 12 (T12) months of treatment. Statistics using repeated measures one-way ANOVA with Tukey’s multiple test. # *p* not significant; **** *p* < 0.0001; ** *p* < 0.01.

In total, 26 T2D patients already in treatment with metformin were recruited and administered with GLP1-RA (namely, liraglutide or dulaglutide) and followed-up for 12 months, with an intermediate visit after 6 months. Clinical and biochemical parameters were collected at baseline visit (T0), and at 6 month (T6) and 12 month (T12) visits (Table 1). As expected, GLP1-RA treatment induced a significant improvement of glycemic control after 6 months of treatment, and a significant weight loss both at 6 and 12 months after treatment (Table 1 and Figure 1A–D). A repeated measures one-way ANOVA was performed to compare the effect of GLP1-RA treatment on several efficacy measures over time, revealing that there was a statistically significant difference in mean HbA1c (*p* = 0.003), BW (*p* = 0.0013) and BMI (*p* = 0.0015) between at least two groups, and a marginally significant difference in FPG (*p* = 0.06). Tukey’s test for multiple comparisons found that the mean value of HbA1c and FPG were significantly different between T0 and T6 (respectively, *p* < 0.0001 and *p* = 0.005), while mean BW and BMI significantly differed between T0 and T6 (respectively *p* = 0.006 and *p* = 0.005) as well as between T0 and T12 (respectively *p* = 0.008 and *p* = 0.009). There was no statistically significant difference in mean values of HbA1c, FPG, BW and BMI between T6 and T12 (Figure 1A–D).

Patients receiving dulaglutide or liraglutide were homogeneous in terms of clinical characteristics at baseline and no difference in treatment outcomes was observed between dulaglutide and liraglutide (Table 2). During the 12 month follow-up, five patients (19.2%) experienced side effects, but these were mostly mild to moderate gastrointestinal events (e.g., nausea, vomiting, constipation, diarrhea), as expected, and only in one case therapy was discontinued. At T6 visit, one patient was lost to follow-up and one patient discontinued treatment to undergo bariatric surgery for morbid obesity. Three patients missed T6 visit due to COVID-19 emergency (visit was converted into a phone contact) but were able to attend T12 visit. At T12 visits, three patients discontinued therapy, in one case due to side effects and in two cases due to treatment failure (defined as failure to reach glycemic target or worsening of glycemic control). One additional patient was lost to follow-up between T6 and T12. No difference in terms of metabolic parameters or anthropometric measures was found between male and female patients (data not shown).

### 2.2. Baseline Levels of the Selected miRNAs Distinguish Patients with Better Treatment Outcomes

Basal levels of the eight circulating miRNAs analyzed (miR-24-3p, miR-126-3p, miR-21-5p, miR-15a-5p, miR-223-3p, miR-378-3p, miR-375-3p and miR-146-5p) did not differ significantly in patients with or without GLP1-RA side effects, nor between patients who discontinued treatment compared to those who continued GLP1-RA. No difference in circulating miRNA levels was observed between males and females.

**Figure 2:**
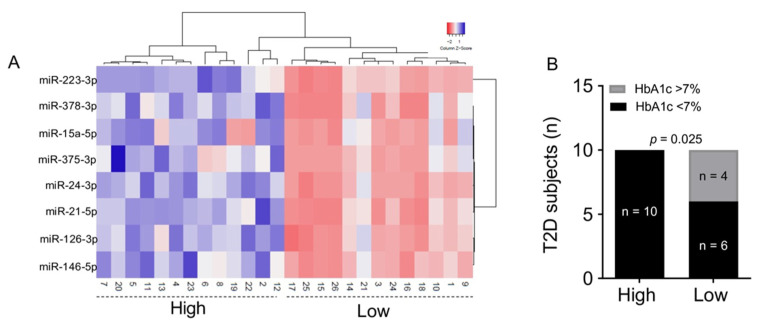
(**A**) Hierarchical clustering dendrogram analysis reporting expression values of each miRNA shown as ∆Ct values and fitted into a color scale (blue: high expression; red: low/null expression) (model: Euclidean−Average linkage); miRNAs are reported in rows while T2D subjects (ID number) are reported in columns. (**B**) Proportion of patients achieving glycemic target in the high expression group and low expression group after 12 months of GLP1-RA therapy. Statistics using Chi-square.

Based on circulating levels of the eight selected miRNAs at baseline, we generated a hierarchical clustering heatmap analysis. T2D subjects were clearly separated into different clusters as shown by column dendrogram. However, color scale expression values representation allowed us to identify two main groups of T2D subjects characterized by high or low global miRNA expression levels, which we defined as “low expressing” (*n* = 13, red color) and “high expressing” miRNAs (*n* = 13, magenta color), as clearly reported in the heatmap (Figure 2A).

In light of the baseline distinct stratification of T2D patients on the basis of candidate miRNAs expression levels, we firstly sought to determine whether the two groups differed at baseline in terms of clinical and biochemical parameters. Metabolic measurements and anthropometric features did not differ between high expressing and low expressing miRNAs groups (Table 3), as well as in the percentage of treatment discontinuation due to side effects. Additionally, the proportion of T2D subjects who experienced one or more side effects was similar between the high and low expressing group.

Of interest, we observed that a significantly higher percentage of patients in the high expressing group achieved the treatment goal (defined as HbA1c < 7%) 12 months after treatment initiation, compared to the low expressing group (*p* = 0.025) (Figure 2B). It is worth noting that based on baseline expression of the selected miRNAs, as shown in Figure 2A, the low expressing group could be further subdivided into two subgroups: very low expressing (*n* = 8, namely subjects 3, 15, 16, 17, 18, 24, 25 and 26) and low expressing (*n* = 5, namely subjects 1, 9, 10, 14 and 21). The difference observed in treatment outcome versus the high expression group is mainly driven by the very low expressing group, in which a significantly lower percentage of patients reached the therapeutic target compared to the remaining patients after 12 months of GLP1-RA treatment (*p* = 0.028) (data not shown).

**Figure 3:**
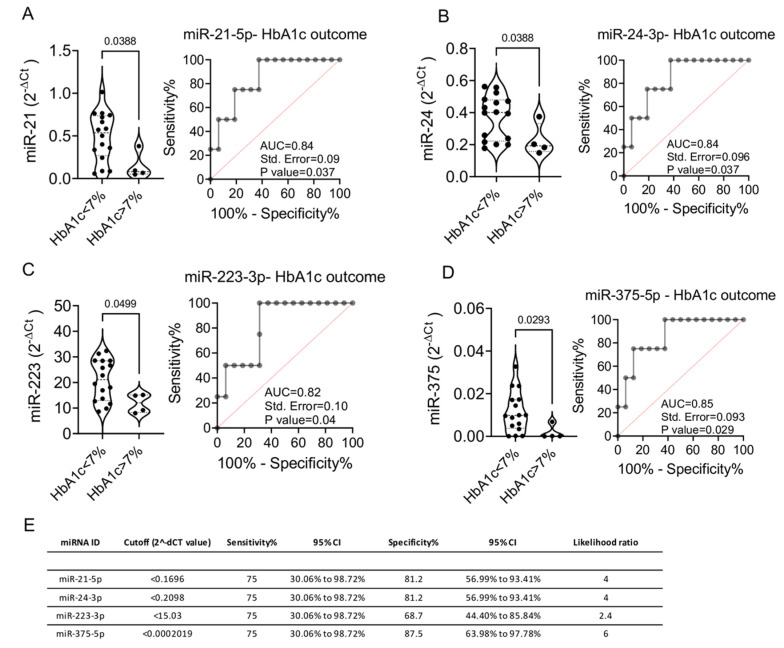
(**A**–**D**) Baseline expression levels of miR-21-5p (**A**), miR-24-3p (**B**), miR-223-3p (**C**) and miR-375-3p (**D**) in patient with HbA1c levels <7% and >7% at T12. ROC curves for each miRNA are reported alongside with indication of Area under the Curve (AUC), Standard Error and *p* value. (**E**) Table reporting baseline miRNAs expression sensitivity and specificity in the prediction of HbA1C > 7% outcome using the ROC curve analysis. Statistics using Mann-Whitney U test for non-parametric data, involving comparisons between two groups.

These data are further supported by the evidence of significantly higher circulating levels of miR-21-5p, miR-24-3p, miR-223-3p and miR-375-5p at baseline in patients who achieved glycemic target at T12 (*p* = 0.03, *p* = 0.03, *p* = 0.04 and *p* = 0.02, respectively) (Figure 3A–D). Receiver operator characteristic (ROC) curves analysis of baseline miRNAs expression levels showed a significant prediction of HbA1c outcome at follow-up for all miRNAs, nevertheless revealing a best fitting model for miR-375-5p with an AUC of 0.85, a specificity of 87.5% and a sensitivity of 75% (Figure 3E). 

**Figure 4:**
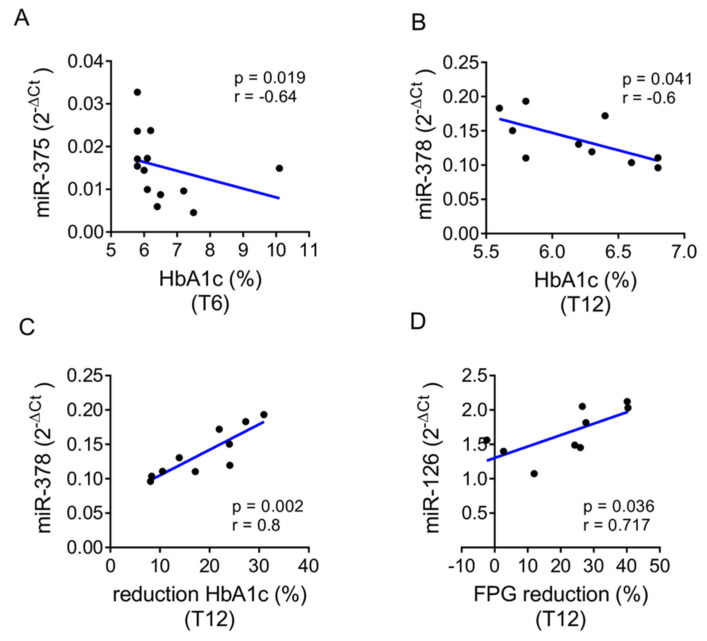
(**A**–**D**) Dot plot graphs of correlations between baseline miRNAs expression levels and metabolic parameters at follow-up in high expressing group. Baseline expression levels of miR-375-3p (**A**) plotted with HbA1c levels assessed at T6, of miR-378a-3p (**B**,**C**) with HbA1c levels and percentage reduction of HbA1c assessed at T12, respectively, and of miR-126-3p (**D**) with percentage reduction of FPG as assessed at T12.

An extended correlation analysis among miRNA expression levels and clinical and biochemical parameters at T0, T6 and T12 was performed including the entire cohort. The low expressing group or the high expressing group of subjects showed several features at T0 with significant correlation to T6 or T12 outcomes. Of particular importance, we observed that in the high expressing group, baseline levels of miR-375-5p and miR-378-3p were inversely correlated with HbA1c levels, respectively, at T6 (*p* = 0.019) and T12 (*p* = 0.041) (Figure 4A,B). Baseline miR-378-3p levels also correlated with HbA1c reduction at T12 with respect to baseline (*p* = 0.002), while miR-126-3p levels were significantly correlated with fasting glucose (FPG) reduction at T12 compared to baseline (*p* = 0.036) (Figure 4C,D). These data suggest that patients with higher baseline expression of miR-378-3p and miR-126-3p have greater reductions in HbA1c and FPG after one year of GLP1-RA therapy.

**Figure 5:**
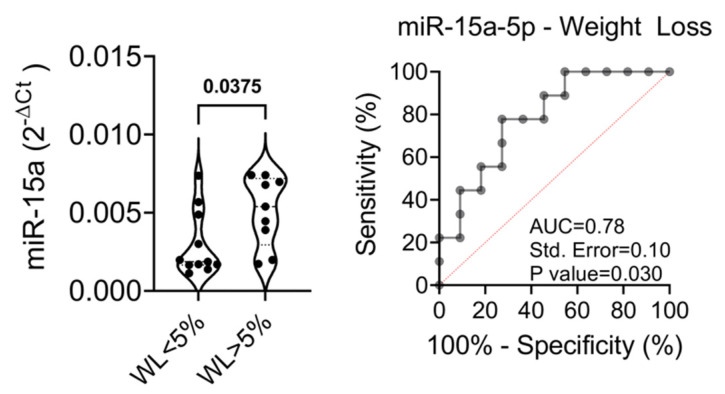
(**Left**) Baseline expression (T0) of miR-15a-5p in patient with WL <5% or >5% at T12. Data are reported as single dot values and mean values of normalized 2^−ΔCt^. Statistics using non-parametric Mann-Whitney U test. FPG: fasting plasma glucose; WL: weight loss. (**Right**) ROC curve of miR-15a-5p baseline expression levels in the prediction of weight loss outcome at 12 months.

The proportion of patients achieving significant weight loss (defined as a loss of at least 5% of the initial weight) was similar in the high and low expressing group (*p* = 0.65); however, patients with >5% weight loss showed significantly higher baseline levels of miR-15a-5p than patients who did not achieve significant weight loss (*p* = 0.03) (Figure 5A,B). The ROC curve analysis showed an AUC of 0.78 with a sensitivity of 77.7% and a specificity of 72.7%, thus revealing that this miRNA could be a good candidate as a predictor of weight loss upon GLP1-RA treatment. 

All the data are included in the present manuscript or shown as supplementary material (Table S1).

## 3. Discussion

T2D is a multifactorial disorder whose prevalence is rapidly increasing worldwide, now being considered a global pandemic and representing a major health and socioeconomic issue worldwide. T2D is associated with high morbidity and mortality; nevertheless, outcomes might be improved by achieving adequate glycemic control early in the course of the disease. However, the achievement of an optimal therapeutic target is often slowed down by the need for several therapeutic attempts before finding the most suitable glucose-lowering therapy for each individual patient. Indeed, despite the availability of abundant treatment options, the percentage of patients with inadequate glycated hemoglobin levels is still unacceptably elevated [43].

Choosing the most appropriate therapy since the disease onset, through the integration of clinical and laboratory data with -omics data, could allow individualized therapy in order to achieve good metabolic balance at an early stage and reduce the risk of treatment failure. Therefore, there is an urgent need to develop novel biomarkers for early diagnosis and prediction of therapeutic response and treatment efficacy to better personalize diabetes treatment [18,20]. Advances in technology, as well as the availability of big data and new analytical tools, provide an intriguing opportunity to make precision medicine part of clinical practice [3,9].

In this scenario, miRNAs have emerged as attractive candidates as diagnostic and predictive biomarkers. Indeed, miRNAs are relatively stable and can be easily detected in biological fluids [18]. Several miRNAs are tissue specific and have been associated to numerous pathological conditions, including diabetes mellitus and its complications [17,18,44]. Accordingly, extensive work on circulating miRNAs in T2D has revealed that many miRNAs are differentially expressed between T2D patients and control subjects [19]. 

Several studies assessed the effects of different antidiabetic treatment on circulating miRNAs expression pattern, with some reporting no effects of therapy on miRNA expression levels [45] and others describing a dysregulation of miRNAs’ expression profiles after treatment initiation [19,20,21,22,46], as well as in patients identified as responders versus non-responders [47]. To the best of our knowledge, there are no data in the literature on the effect of metformin or GLP1-RA treatment on the expression levels of the eight miRNAs we investigated, with the exception of miR-24-3p, 21-5p, miR-146a-3p and miR-126-3p, which, alongside with other miRNAs, were found to be downregulated after 3 months of metformin treatment in the study by Demirsoy and colleagues [19].

Data on the potential use of miRNAs in clinical practice as predictive tools to guide the selection of anti-hyperglycemic drugs are still scarce. In this study, we aimed at finding a panel of circulating miRNAs to be used for a priori assessment of treatment efficacy. To this end, we analyzed baseline expression of eight selected miRNAs in the plasma of type 2 diabetic patients starting treatment with GLP1 analogues. To our knowledge, this is the first prospective study to investigate miRNA expression in GLP1-RA treated diabetic patients.

Our data showed that a panel of eight circulating miRNAs might represent an interesting tool to identify diabetic subjects who will benefit from GLP1-RA treatment and are most likely to achieve the therapeutic target. In particular, according to our data, patients with higher baseline levels of miR-21-5p, miR-24-3p, miR-223-3p and miR-375-3p have a better glycemic outcome after one year of GLP1-RA therapy, while higher baseline expression of miR-378-3p and miR-126-3p is associated with significant reductions in HbA1c and fasting blood glucose at follow-up. In addition, subjects with higher baseline levels of miR-15a-5p achieved significant weight loss after 1 year of treatment.

Such miRNAs have already been associated with glucose metabolism and β-cell function, as well as to obesity and cardiovascular diseases. Differential expression of these miRNAs has been reported in patients with obesity and/or T2D. For instance, upregulation of miR-223-3p is critical for maintaining functional β-cell mass, by promoting β-cell proliferation and improving β-cell function [48] and has been recently linked to preadipocyte dysfunction associated with T2D development [41]. Available data support the role of miR-146a-5p in suppressing obesity-associated inflammation and protecting against obesity-induced high-fat diet [49,50]. Reduced miR-146a-5p levels have been found in obese T2D patients and the loss of miR-146a-5p has been associated to significant weight gain and impaired glycaemia upon high-fat diet-fed mice [49].

Among the selected miRNAs, miR-15-5p, miR-375-3p and miR-21-5p are associated to insulin production in pancreatic β-cells. A significant downregulation of miR-15a-5p has been demonstrated in pre-diabetes and T2D, representing a useful tools to predict the onset of the disease and to differentiate subjects with alteration of glycemic metabolism from healthy controls [51]. Moreover, plasma levels of miR-15a-5p correlate with disease severity [52]. Among β-cell-specific miRNAs, miR-375 has also been related to β-cell damage/proliferation and to insulin secretion, and is involved in the maintenance of islet architecture [44,50]. In more detail, miR-375 leads to a reduced glucose-stimulated insulin secretion by inhibiting exocytosis and downregulating insulin expression [50]. MiR-378a has been associated to lipid storage and metabolism, and mitochondrial function [53]. Furthermore, miR-378 has been shown to be a rheostat of glucose and lipid homeostasis and, indeed, hepatic miR-378 is a core regulator of insulin signaling [54]. MiR-21 has been associated with pancreatic islets inflammation and also promotes adipocyte differentiation [50]. Finally miR-24-3p has been attributed a protective role against apoptosis during metabolic stress [55].

Intriguingly, the association of these miRNAs to both diabetes and cardiovascular disease is particularly interesting in view of the well-known cardiovascular protective effect of GLP-1 analogues in diabetic patients. Recently, miR-126-3p and miR-223-3p have been suggested as biomarkers of ischemic risk stratification after myocardial infarction. Indeed, miR-223-3p level was significantly related to an increased risk of ischemic events and the miR-126-3p to miR-223-3p ratio was related to a decreased risk of occurrence [56]. It has previously been shown that miR-126-3p significantly discriminates between subjects with and without cardiovascular diseases, and correlates with several glycemic and lipid indices [31]. Downregulation of miR-126-3p plays a pathogenic role in the development of diabetic complications, consistent with its role in endothelial homeostasis, angiogenesis and vascular integrity [23,57]. Furthermore, Zampetaki et al. have shown that miR-126-3p is downregulated in T2D, preceding disease onset and thus representing an optimal candidate biomarker [23]. A study by Deng and colleagues demonstrated that levels of circulating miR-24-3p were also able to distinguish between T2D patients with cardiovascular disease from both non-diabetic patients with cardiovascular disease and control subjects [58].

Further data are needed to clarify the relationship between miRNAs expression and the mechanisms underlying the onset of diabetes, obesity and cardiovascular disease. Our data suggest that the assessment of specific circulating miRNAs prior to initiating GLP1-RA therapy, in combination with clinical and metabolic parameters usually collected in clinical practice, may allow an estimation of treatment outcome in terms of glycated hemoglobin reduction and weight loss, in order to guide the choice of therapy.

The main limitation of our study is the small sample size. However, we believe that the results presented, although obtained in a small cohort of patients, may be of interest in the field of precision medicine, stimulating further investigation on this topic, not sufficiently investigated to date. Another potential limitation is represented by the lack of data regarding the correlation of the selected miRNAs with adipokines or markers of insulin resistance. Indeed, insulin dosing is not performed on a routine basis in our outpatients. Similarly, we did not measure the levels of pivotal adipokines in clinical practice. 

To our knowledge, this is the first study to demonstrate a role for miRNAs as potential predictive biomarkers of therapeutic response to GLP1-RA. In our opinion, this pilot study, although needing validation on a larger sample size to strengthen the results obtained, is highly encouraging, paving the way for precision medicine in diabetes. 

## 4. Materials and Methods

### 4.1. Patients

In total, 26 T2D patients (9 females and 17 males; mean age ± SD 60.3 ± 10.3 years, range 35–79; mean disease duration ± SD 10.2 ± 8.7 years), affected by T2D and followed at Diabetes Unit of University of Siena (Italy), were evaluated. All patients were already treated with metformin and did not meet the glycemic target (mean HbA1c± SD = 7.7 ± 0.58%). After initial full metabolic assessment, patients who met criteria for GLP1-RA therapy were selected to receive dulaglutide (*n* = 18) or liraglutide (*n* = 8). Patients were evaluated before (T0) and after six (T6) and twelve (T12) months after starting GLP1-RA. Patients were instructed to titrate liraglutide from 0.6 mg/day to 1.2 mg/day after a week and to 1.8 mg/day after another week, if necessary to achieve desired goal, unless contraindicated or not tolerated; dulaglutide was started at a dose of 1.5 mg/week unless contraindications or intolerance. Patient characteristics are shown in Table 1. 

### 4.2. Clinical and Biochemical Parameters

At each follow-up visit, treatment efficacy was evaluated through clinical examination including body weight measurement, and blood exams, including HbA1c, fasting plasma glucose, urinalysis, renal and hepatic function and lipid profile. Information on side effects, self-monitoring blood glucose and treatment compliance were also collected.

### 4.3. Blood Collection Procedure

Plasma was obtained from venous blood and processed according to a standardized procedure [59]. Briefly, blood was collected in BD Vacutainer K_2_-EDTA tubes (BD Biosciences), inverted 5 times and stored upright at room temperature (18–25 °C) until ready for processing. Blood samples were processed within 2 h from blood draw by centrifugation at 1800× *g* for 10 min at room temperature; collected plasma was further centrifuged at 1200× *g* for 20 min at 10 °C in order to remove contaminant cells, platelets and cell debris. Finally, plasma samples were aliquoted (200 μL each aliquot) in order to avoid repeated freeze-thaw cycles and subsequently stored at −80 °C until further use.

### 4.4. RNA Extraction from Plasma Samples

RNA was extracted from 200 μL of plasma using on-column RNA extraction by adopting Serum/Plasma RNA Purification Kit (Norgen). Frozen plasma samples were thawed on ice and then further centrifuged 2′ at 400g to remove eventual residual cell debris and to avoid column clogging. On-column RNA extraction was then performed following manufacturer’s recommendations. Finally, RNA was eluted in 20 μL of nuclease-free water and stored at −80 °C until further use.

### 4.5. Circulating microRNAs qRT-Real-Time PCR Analysis

The expression of miR-24-3p, miR-21-5p, miR-146a-5p, miR-375-3p, miR-126-3p, miR-15a-5p, miR-378a-3p and miR-223-3p was analyzed in all 26 plasma samples through single assay qRT Real-Time PCR using TaqMan miRNA assay primers (Lifetechnologies, Carlsbad, CA, USA) as previously reported [53]. A total of 3 μL of RNA was reverse-transcribed employing Custom RT primers pool and preamplified using Custom Preamp primers pool. Briefly, 5 µL each RT or TM primer was diluted in a total volume of 500 µL TE1X and used for RT or Preamplification reaction. Then, 3 μL of extracted RNA was added to 6 μL custom primers pool, 0.30 μL 100 mM dNTPs, 3 μL of 50 U/μL Multiscribe RT, 1.50 μL 10× RT Buffer, 0.19 μL 20 U/μL RNAse Inhibitor and 1.01 μL H_2_O. The reaction product was incubated at 16 °C for 30 min, 42 °C for 30 min and then at 85 °C for 5 min. Afterwards, the synthesized cDNA was preamplified using Custom Preamp primer pool: 2.5 μL cDNA from each sample was added to 12.5 μL 2× TaqMan Preamp Master Mix, 3.75 μL 10× Custom Preamp primers and 6.75 μL H2O. It was incubated at 95 °C for 10 min, at 55 °C for 2 min and at 72 °C for 2 min, then for 12 cycles at 95 °C for 15 s and 60 °C for 4 min and, finally, at 99 °C for 10 min. In each well, 5 μL preamplified cDNA (diluted 1:8) was added to 15 μL reaction mix composed of 10 μL TaqMan Universal Master Mix, 1 μL TaqMan miRNA expression assay, 4 μL nuclease-free H_2_O. The reaction was incubated at 95 °C for 10 min, followed by 40 cycles at 95 °C for 15 s and at 60 °C for 1 min. MiRNAs expression data analysis was analyzed using 2^−ΔCt^ method, using miR-191-5p as endogenous control (normalizer), previously shown to be stably expressed in plasma/serum samples in multiple context and studies, thus optimal as a circulating housekeeping miRNA. Samples with resulting raw cycle-threshold (Ct) > 35.0 were considered as not detected/expressed.

### 4.6. Statistics

Statistical analysis was performed with GraphPad Prism version 8.1.1 and 9 (GraphPad Prism, La Jolla, CA, USA). Repeated measures one-way ANOVA was used to analyze group differences over different timepoints, and Tukey’s multiple comparisons test was used to calculate differences between groups. Mann-Whitney U test for non-parametric data involving comparisons between two groups, or Wilcoxon test and Kruskal-Wallis with Dunn’s post-test were used to determine the differences between groups/samples. To compare variables among categories, Chi-square test was used. Correlation test used was linear regression analysis using Spearman R test. All data are presented as single dot values and mean value. A *p*-value < 0.05 was considered significant.

## Figures and Tables

**Table 1:** Anthropometric measures and metabolic data of diabetic patients at baseline (T0) and after 6 (T6) and 12 (T12) months of GLP1-RA therapy.

	T0 (*n* = 26)	T6 (*n* = 21)	T12 (*n* = 20)
Age (years)	60.3 ± 10.3 (35–79)	/	/
Disease Duration (years)	10.2 ± 8.7 (1–32)	/	/
Weight (kg)	95.6 ± 20.6 (56–134.7)	92.9 ± 20.2 (58–133) *	93.3 ± 17.8 (51–121) §
BMI (kg/m^2^)	33.7 ± 6.5 (21.9–49.5)	32.0 ± 5.9 (23.2–48.5) *	32.4 ± 4.9 (19.9–41.9) §
HbA1c (%)	7.7 ± 0.58 (6–8.8)	6.4 ± 1.0 (5.3–10.1) *	6.7 ± 1.7 (5.5–13.2)
FPG (mg/dL)	159.1 ± 27.9 (112–223)	128.9 ± 37.5 (86–251) *	134.7 ± 56.3 (91–355)
Total Cholesterol (mg/dL)	157.5 ± 42.4 (54–258)	155.6 ± 26.5 (106–208)	150.2 ± 24.8 (104–200)
Triglycerides (mg/dL)	138.7 ± 63.1 (56–313)	129.9 ± 49.3 (60–257)	132.6 ± 58.5 (61–232)
HDL (mg/dL)	46.3 ± 11.7 (28–72)	44.9 ± 10.9 (28–71)	46.5 ± 11.8 (29–76)
LDL (mg/dL)	92.2 ± 34.9 (50–173)	84.7 ± 22.6 (49–130)	76.2 ± 24.1 (45–131)
Creatinine (mg/dL)	0.91 ± 0.23 (0.64–1.34)	0.93 ± 0.19 (0.67–1.27)	0.93 ± 0.26 (0.59–1.56)
eGFR (mL/min/1.73 m^2^)	84.7 ± 21.1 (44–116)	85.0 ± 17.4 (47–112)	84.3 ± 21.5 (37–122)
Albuminuria (mg/dL)	46.0 ± 104.4 (0–462)	199.0 ± 681.2 (0–2834)	73.8 ± 171.4 (0–575)
%WL	/	4.9 ± 5.8 (−5.2–23.3)	5.5 ± 6.1 (−2.9–18.1)
%EWL	/	18.9 ± 26.8 (−41.2–81.2)	15.5 ± 16.4 (−9.7–50)
HbA1c reduction (%)	/	16.5 ± 14.4 (−31.2–36.9)	12.7 ± 22.3 (−69.2–33.3)

Data are expressed as mean ± SD, range is shown in brackets. BMI: body mass index; FPG: fasting plasma glucose; HDL: high density lipoprotein; LDL: low density lipoprotein; eGFR: estimated glomerular filtration rate, calculated by CKD-EPI (Chronic Kidney Disease Epidemiology Collaboration) equation; %WL: percentage of weight loss; %EWL: percentage of excess weight loss. * *p* < 0.05 at T6 versus T0; § *p* < 0.05 at T12 versus T0 (statistics using one-way ANOVA for repeated measures with Tukey’s multiple comparisons test).

**Table 2:** Anthropometric measures and metabolic data in patients treated with dulaglutide or liraglutide, before and after therapy.

	Dulaglutide			Liraglutide			*p* Value
	T0 (*n* = 18)	T6 (*n* = 14)	T12 (*n* = 13)	T0 (*n* = 8)	T6 (*n* = 7)	T12 (*n* = 7)	
Age (years)	60.1 ± 12.1 (35–79)	/	/	61.0 ± 4.9 (55–67)	/	/	* 0.83
Disease Duration (years)	10.8 ± 9.4 (1–32)	/	/	8.8 ± 7.3 (1–23)	/	/	* 0.65
Weight (kg)	93.6 ± 19.3 (56–127)	91.4 ± 17.6 (58–124)	93.4 ± 17.6 (51–119)	100.0 ± 24.2 (72–134.7)	95.8 ± 26.0 (65.5–133)	93.4 ± 19.5 (70–121)	* 0.61; § 0.87; # 0.8
BMI (kg/m^2^)	32.5 ± 5.1 (21.9–41)	32.4 ± 4.5 (23.5–40.0)	32.4 ± 4.6 (19.9–40.2)	36.3 ± 8.7 (25.5–49.5)	33.3 ± 8.5 (23.2–48.9)	32.4 ± 5.8 (24.8–41.9)	* 0.53; § 0.91; # 0.81
HbA1c (%)	7.6 ± 0.6 (6.0–8.4)	6.6 ± 1.2 (5.3–10.1)	6.2 ± 0.6 (5.5–7.4)	7.8 ± 0.6 (7.0–8.8)	6.2 ± 0.6 (5.7–7.5)	7.5 ± 2.6 (5.8–13.2)	* 0.77; § 0.42; # 0.23
FPG (mg/dL)	156.9 ± 30.9 (112–223)	133.7 ± 41.4 (86–251)	120.2 ± 14.4 (94–141)	164.0 ± 20.7 (143–201)	119.3 ± 28.4 (92–176)	166.0 ± 95.8 (91–355)	* 0.45; § 0.36; # 0.23
Total Cholesterol (mg/dL)	145.9 ± 36.8 (54–220)	157.7 ± 27.7 (106–208)	144.8 ± 18.7 (112–178)	182 ± 45.3 (123–258)	151.6 ± 25.5 (119–187)	161.8 ± 33.8 (104–200)	* 0.1; § 0.57; # 0.14
Triglycerides (mg/dL)	138.1 ± 68.1 (56–313)	129.9 ± 56.0 (60–257)	120.8 ± 55.0 (61–222)	140.0 ± 55. (77–229)	129.7 ± 37.8 (88–187)	163.2 ± 61.9 (110–232)	* 0.74; § 0.89; # 0.11
HDL (mg/dL)	46.3 ± 12.8 (28–72)	44.8 ± 11.9 (28–71)	48.5 ± 13.0 (31–76)	46.4 ± 9.6 (30–61)	45.1 ± 9.6 (28–57)	42.5 ± 8.3 (29–52)	* 0.76; § 0.86; # 0.43
LDL (mg/dL)	83.0 ± 29.3 (51–147)	87.0 ± 22.9 (54–130)	71.9 ± 20.7 (47–110)	114.5 ± 39.44 (50–173)	80.5 ± 23.0 (49–106)	86.4 ± 31.1 (45–131)	* 0.08; § 0.65; # 0.31
Creatinine (mg/dL)	0.94 ± 0.2 (0.64–1.34)	0.96 ± 0.2 (0.67–1.27)	0.94 ± 0.3 (0.59–1.56)	0.9 ± 0.2 (0.64–1.23)	0.87 ± 0.2 (0.67–1.06)	0.89 ± 0.3 (0.66–1.37)	* 0.5; § 0.52; # 0.61
eGFR (mL/min/1.73 m^2^)	85.1 ± 23.9 (44–116)	84.5 ± 20.9 (47–112)	84.5 ± 23.7 (37–122)	83.8 ± 14.1 (61–97)	86.0 ± 9.3 (72–98)	83.6 ± 16.7 (54–93)	* 0.69; § 0.83; # 0.75
Albuminuria (mg/dL)	51.6 ± 120.3 (0–462)	314.0 ± 887.5 (0–2834)	88.9 ± 187.9 (0− 575)	30.5 ± 39.6 (4–97.6)	34.9 ± 51.3 (3.2–143)	6.0 ± 1.4 (5–7)	* 0.89; § 0.98; # 0.54
%WL	/	5.0 ± 6.9 (−5.3–23.3)	5.3 ± 6.3 (−2.9–18.1)	/	4.7 ± 3.2 (1.3–9.0)	5.8 ± 6.2 (−1.9–15.4)	§ 0.74; # 0.81
%EWL	/	17.2 ± 27.7 (−41.2–64.3)	14.3 ± 18.5 (−9.7–50)	/	22.5 ± 26.7 (2.3–81.2)	17.5 ± 13.4 (−6.5–34.1)	§ 0.7; # 0.53
HbA1c reduction (%)	/	14.8 ± 16.3 (−31.2–36.9)	18.0 ± 11. (−8.3–33.3)	/	19.8 ± 9.8 (3.8–35.2)	2.9 ± 33.5 (−69.2–29.5)	§ 0.59; # 0.35

Data are expressed as mean ± SD, range is shown in brackets. BMI: body mass index; FPG: fasting plasma glucose; HDL: high density lipoprotein; LDL: low density lipoprotein; eGFR: estimated glomerular filtration rate, calculated by CKD-EPI (Chronic Kidney Disease Epidemiology Collaboration) equation; %WL: percentage of weight loss; %EWL: percentage of excess weight loss. Statistics using non-parametric Mann-Whitney U Test. Comparison between dulaglutide and liraglutide at T0 (*), T6 (§) and T12 (#).

**Table 3:** Anthropometric measures and metabolic data in low expressing group and high expressing group.

	Low Expressing (*n* = 13)			High Expressing (*n* = 13)			*p* Value
	T0	T6	T12	T0	T6	T12	
Age (years)	59.5 ± 10.7 (35–74)	/	/	61.9 ± 10.1 (41–79)	/	/	* 0.65
Disease Duration (years)	9.3 ± 10.5 (1–32)	/	/	11.1 ± 6.7 (2–23)	/	/	* 0.24
Weight (kg)	97.3 ± 19.7 (56–129)	97.4 ± 17.1 (74–124)	93.3 ± 20.8 (51–121)	93.9 ± 22.2 (64–134.7)	90.1 ± 22.1 (58–133)	93.4 ± 15.4 (70–114)	* 0.65 § 0.3 ¶ 0.69
BMI (kg/m^2^)	34.1 ± 6.3 (21.8–46.6)	32.1 ± 4.2 (26.3–38.3)	32.1 ± 5.3 (19.9–40.2)	33.3 ± 6.9 (25.5–49.5)	31.9 ± 6.9 (23.2–48.8)	32.8 ± 4.7 (24.8–41.9)	* 0.67 § 0.74 ¶ > 0.9
HbA1c (%)	7.7 ± 0.7 (6–8.8)	6.2 ± 0.75 (5.3–7.5)	7.1 ± 2.3 (5.5–13.2)	7.7 ± 0.4 (7–8.4)	6.7 ± 1.2 (5.8–10.1)	6.2 ± 0.4 (5.6–6.8)	* 0.24 § 0.5 ¶ 0.4
FPG (mg/dL)	160.4 ± 32.0 (112–201)	123.0 ± 30.3 (86–176)	146.5 ± 75.5 (94–355)	157.8 ± 24.5 (128–223)	132.5 ± 42.0 (93–251)	121.6 ± 17.5 (91–145)	* 0.62 § 0.76 ¶ 0.7
Total Cholesterol (mg/dL)	148.6 ± 49.3 (54–258)	147.3 ± 31.4 (106–187)	143.8 ± 23.7 (104–190)	167.1 ± 32.7 (124–220)	160.0 ± 23.6 (131–208)	157.3 ± 25.4 (112–200)	* 0.17 § 0.3 ¶ 0.19
Triglycerides (mg/dL)	140.0 ± 72.6 (73–313)	151.4 ± 61.8 (72–257)	127.0 ± 72.5 (54–232)	137.3 ± 54.1 (56–245)	118.2 ± 39.0 (60–188)	129.3 ± 48.6 (62–219)	* 0.75 § 0.3 ¶ 0.56
HDL (mg/dL)	47.6 ± 11.5 (30–68)	42.4 ± 10.2 (28–54)	56.7 ± 31.7 (29–136)	45.1 ± 12.3 (28–72)	46.2 ± 11.4 (28–71)	45.4 ± 12.8 (31–76)	* 0.5 § 0.49 ¶ 0.49
LDL (mg/dL)	89.7 ± 40.3 (51–173)	74.7 ± 19.6 (49–99)	68.9 ± 18.9 (45–96)	94.8 ± 30.2 (50–149.8)	90.9 ± 23.7 (55.4–130)	84.9 ± 28.4 (47–131)	* 0.47 § 0.29 ¶ 0.86
Creatinine (mg/dL)	0.94 ± 0.26 (0.64–1.34)	0.96 ± 0.18 (0.67–1.27)	0.98 ± 0.31 (0.59–1.56)	0.88 ± 0.21 (0.67–1.34)	0.90 ± 0.19 (0.67–1.24)	0.85 ± 0.18 (0.66–1.19)	* 0.69 § 0.46 ¶ 0.28
eGFR (mL/min/1.73 m^2^)	83.1 ± 25.0 (44–116)	83.9 ± 20.4 (47–113)	81.7 ± 26.8 (37–123)	85.9 ± 16.9 (56–111)	84.3 ± 18.1 (57–112)	88.4 ± 13.9 (65 – 110)	* 0.9 § 0.95 ¶ 0.7
Albuminuria (mg/dL)	81.1 ± 157.1 (0–462)	534.3 ± 1130 (2–2834)	128.5 ± 225.4 (5–575)	20.5 ± 25.0 (2–67)	16.1 ± 17.9 (0–57)	8.2 ± 9.68 (0–23)	* 0.59 § 0.2 ¶ 0.09
%WL	/	6.36 ± 7.2 (0.86–23.3)	4.4 ± 6.1 (−1.9–18.1)	/	14.2 ± 15.7 (−31.2–29.3)	6.5 ± 6.2 (−2.8–15.4)	§ 0.79 ¶ 0.4
%EWL	/	22.9 ± 23.2 (3.2–64.3)	11.5 ± 17.7 (−7.8–50)	/	16.5 ± 29.5 (−41.2–81.2)	19.0 ± 15.1 (−9.7–42.4)	§ 0.68 ¶ 0.3
HbA1c reduction (%)	/	20.2 ± 12.0 (3.85–36.9)	6.8 ± 30.1 (−69.2–33.3)	/	14.2 ± 15.7 (−31.2–29.3)	18.6 ± 8.2 (8.1–30.9)	§ 0.69 ¶ 0.48

Data are expressed as mean ± SD, range is shown in brackets. Statistics using non-parametric Mann-Whitney U Test. BMI: body mass index; FPG: fasting plasma glucose; HDL: high density lipoprotein; LDL: low density lipoprotein. eGFR: estimated glomerular filtration rate, calculated by CKD-EPI (Chronic Kidney Disease Epidemiology Collaboration) equation; %WL: percentage of weight loss; %EWL: percentage of excess weight loss. Statistics using non-parametric Mann-Whitney U Test. * comparison between low expressing and high expressing patients’ parameters at T0 (*), T6 (§) and T12 (¶).

## Data Availability

All the data are included in the present manuscript or shown as supplementary material.

## Supplementary Materials

The following are available online at https://www.mdpi.com/article/10.3390/ijms22179454/s1.
