# Supplementary material for: Circulating microRNAs Signature for Predicting Response to GLP1-RA Therapy in Type 2 Diabetic Patients: A Pilot Study
Source: Int J Mol Sci. 2021 Aug 31;22(17):9454. doi: 10.3390/ijms22179454 (PMC8431190; doi:10.3390/ijms22179454)
Supplement: Supplementary file 1 [file ijms-22-09454-s001.zip › ijms-1329307-supplementary.pdf]

Table S1. Baseline expression levels of the 8 miRNAs analyzed (reported as $2^{-\Delta Ct}$).

	MicroRNAs Expression ($2^{-\Delta Ct}$)							
**Sample ID**	**miR-24**	**miR-126**	**miR-15a**	**miR-21**	**miR-223**	**miR-378**	**miR-375**	**miR-146**
1	0.2165841	0.86154607	0.00170029	0.2443466	12.7021413	0.03472216	0.00499604	0.68586635
2	0.52413153	1.81503908	0.00697303	1.0139598	19.4675705	0.19317767	0.00875305	1.50733591
3	0.20306321	0.72698672	0.00198589	0.09486378	14.9078543	0.01131987	0.00019301	0.52924247
4	0.50557563	2.07627703	0.00537694	0.74638873	26.1184762	0.16770525	0.01490561	1.20330319
5	0.39914898	2.05053348	0.00740645	0.76260043	28.5021764	0.18301062	0.0172531	1.33514813
6	0.37474981	1.72070639	0.00691526	0.63463538	35.3343545	0.09875515	0.004534	0.85322604
7	0.48263331	1.56048911	0.00567958	0.58479376	28.4626755	0.11064417	0.00961165	1.34164203
8	0.39447368	1.48761372	0.00740646	0.7194666	31.2763654	0.1504129	0.00593721	0.80496629
9	0.23439271	0.98486688	0.00522628	0.35111116	12.9780341	0.08573329	0.0113907	0.50592667
10	0.25863686	0.95197847	0.00487967	0.35998307	13.6895168	0.09525904	0.00990245	0.55171695
11	0.58560458	1.82007565	0.00755161	0.73969305	28.8800089	0.07499814	0.01540988	1.51361634
12	0.40304172	2.0307313	0.00736551	0.742777	18.0634448	0.172062	0.02361751	0.83682626
13	0.43107156	1.07400495	0.00301004	0.76577888	26.6858095	0.10373689	0.02376529	0.98965708
14	0.23898624	1.01818454	0.00445302	0.33657477	16.9474731	0.05303169	0.00343613	0.55478459
15	0.18301062	0.59460356	0.00169206	0.04866404	9.18957955	0.00176759	6.0404E-05	0.37683348
16	0.20152059	0.6620439	0.00198451	0.08838835	11.6479149	0.00239626	5.5853E-05	0.3285077
17	0.19971306	0.35135495	0.00172762	0.08549594	9.82188874	0.00556655	0.00016491	0.48937078
18	0.17218124	0.56644131	0.00162652	0.05987094	10.867886	0.00294401	0.00010365	0.59542797
19	0.47171925	1.45296652	0.00187486	0.50208395	32.3344754	0.11957609	0.00995751	0.93432762
20	0.4613715	1.39958646	0.00677765	0.57236272	28.5615118	0.09612132	0.03271282	1.03168323
21	0.37500951	1.44693392	0.00389813	0.38024457	15.1579104	0.11414974	0.00682005	0.86693679
22	0.55709688	2.12284426	0.00165609	0.4008126	22.7689976	0.11033785	0.01444786	1.16958753
23	0.56252927	1.54756614	0.00538812	0.66296357	25.7410826	0.13057945	0.01708649	1.63240692
24	0.20222037	0.75996248	0.00162539	0.25243781	15.7797183	0.04337476	0.0011025	0.51121412
25	0.14885699	0.47204615	0.00138203	0.06656924	8.03891039	0.00216713	1.3954E-05	0.33056362
26	0.17751348	0.56136064	0.00112645	0.05839548	8.66380472	0.00307116	0.00021078	0.36475511
